# Patient's care bundle benefits to prevent stroke associated pneumonia: A meta-analysis with trial sequential analysis

**DOI:** 10.3389/fneur.2022.950662

**Published:** 2022-10-31

**Authors:** Feng Guo, Qiao Fan, Xiaoli Liu, Donghai Sun

**Affiliations:** ^1^Department of Emergency Center, Xi'an International Medical Center Hospital, Xi'an, China; ^2^Department of Intensive Care Unit, Xi'an International Medical Center Hospital, Xi'an, China; ^3^Department of Imaging, Xi'an Central Hospital, Xi'an, China

**Keywords:** stroke, pneumonia, care bundle, meta-analysis, trial sequential analysis

## Abstract

**Background:**

Patient's care bundle has been found to have a beneficial effect on refractory diseases, but the preventive effect of this strategy on stroke-associated pneumonia (SAP) remains unclear. The purpose of this meta-analysis was to determine the role of the patient's care bundle in the prevention of SAP.

**Methods:**

A systematic search was conducted in five electronic databases to identify randomized controlled trials (RCTs) published before January 31, 2022. The incidence of SAP and aspiration and the length of hospital stay were assessed. Random pair-wise meta-analysis was conducted using Review Manager 5.4, and trial sequential analysis (TSA) was also performed.

**Results:**

Twenty eligible RCTs involving 1916 patients were included for data analysis. Pooled results suggested that patient's care bundle was associated with significantly lower incidence of SAP (risk ratio [RR], 0.37; 95% CI, 0.29–0.46; *p* < 0.001; I^2^ = 0%) and aspiration (RR, 0.23; 95% CI, 0.15–0.35; *p* < 0.001; I^2^ = 0%). Meanwhile, patient's care bundle also significantly shortened the length of hospital stay for general patients (mean difference [MD], −3.10; 95% CI, −3.83 to −2.37; *p* < 0.001; I^2^ = 16%) and the length of intensive care unit (ICU) stay for patients with severe stoke (MD, −4.85; 95% CI, −5.86−3.84; *p* < 0.001; I^2^ = 0%). Results of TSA confirmed that none of the findings could be significantly reversed by future studies.

**Conclusions:**

The patient's care bundle effectively prevents the occurrence of SAP and aspiration and shortens the hospital stay of stroke patients. However, it is necessary to design more high-quality studies to further validate our findings and investigate their applicability in other geographical regions.

## Background

Stroke-associated pneumonia (SAP) refers to new-onset pneumonia within the first week after stroke (1–3), and was first defined by Hilker et al. (4). As one of the most common complications in stroke patients, the incidence of SAP is 6.7% to 37.98% (5–8). SAP is considered an important risk factor for death after stroke because stroke patients have a worse prognosis if SAP is diagnosed (9). Studies have shown that the presence of SAP is associated with increased risk of morbidity, increased 30-day incidence, and increased 1-year mortality, making patients experience an extended length of hospitalization and more medical expenditures (10–12). Notably, pneumonia has been shown to significantly increase the risk of multiple non-pneumonic medical complications in patients with AIS, such as gastrointestinal bleeding and recurrent stroke (7). Therefore, it's essential to prevent the occurrence of SAP (13).

Nursing interventions play a significant role in the comprehensive management of stroke patients (14). Traditionally, routine care interventions are implemented daily for caring for stroke patients based on clinical experience; however, it cannot fully address all the problems faced by stroke patients due to several inherent problems (15, 16). Recently, patient's care bundle has been widely used to manage refractory conditions in clinical practice (17, 18). Previously published studies have evidenced that the application of the care bundle is associated with a lower incidence of ventilator-associated pneumonia and central line-associated bloodstream infection (19, 20). Meanwhile, studies have also shown that the care bundle may prevent the development of SAP and improve the functional outcomes in patients with ischemic stroke (21).

Currently, the components of the care bundles for SAP preventions varied in literatures; however, four major aspects were consistently involved in the currently available bundles, including feeding management, body position management, oral care, and respiratory tract management (22). Nevertheless, several randomized controlled trials (RCTs) (23–26) with limited sample sizes have been conducted to investigate the interventional effect of care bundle on the prevention of SAP in stroke patients; however, these studies have reported conflicting results. However, the exact role of the care bundle in preventing SAP in stroke patients remains unclear. Therefore, we performed this meta-analysis with the trial sequential technique to systematically determine the role of the care bundle in preventing SAP in stroke patients.

## Materials and methods

We designed this meta-analysis following the recommendations of the Cochrane handbook for systematic review (27), although no formal protocol was publicly reported. In addition, we reported pooled results according to the Preferred Reporting Items for Systematic Reviews and Meta-Analyses statement (28). Certainly, as a meta-analysis of published studies, ethical approval and informed consent from patients are not required.

### Study identification

We systematically searched PubMed, EMBASE, the Cochrane Library, China National Knowledge Infrastructure (CNKI), Wanfangdata, and the Chinese Biological Medical Database (Sinomed) for potentially eligible randomized controlled trials (RCTs) from inception until January 31, 2022. Two investigators (Feng Guo and Qiao Fan) independently conducted literature searches, and literature searches were unrestricted. The following terms were used to develop the search strategies using the medical subject heading (MeSH) in combination with text terms: (“stroke” or “cerebrovascular accident” or “brain vascular accident” or “apoplexy”) and (“pneumonia” or “pneumonitis”) and (“care bundle” or “cluster care” or “intensive nursing”). Supplementary Table S1 summarizes the full search strategies for three English databases. We also screened a reference list of eligible studies to search for additional studies. Any disagreements were resolved by consulting a third senior investigator (Donghai Sun).

### Study selection

We developed a literature database by importing retrieved records into EndNote software, and then automatically deduplicated records. Eligibility was assessed based on screening of title, abstract, and full-text. Briefly, the following inclusion criteria were used to evaluate the eligibility for each study: (a) adult stroke patients verified by CT or MRI within 24 h of admission; (b) patients were managed with the care bundle or routine care protocols; (c) studies reported incidence of SAP and aspiration and the length of hospital stay; and (d) studies were reported as RCTs with full-texts.

We excluded studies if they met at least one of the following criteria: (a) patients were confirmed to have SAP before assessment; (b) studies were repeated reported; (c) conference abstracts with insufficient data; and (d) essential data that cannot be supplemented after contacting the corresponding author were missing.

### Data extraction

Two independent investigators (Qiao Fan and Xiaoli Liu) extracted basic information from each study using the standard data extraction sheet. Specifically, the following data were extracted: the basic information of references (the first author's name, year of publication, and country), the baseline characteristics of patients (sample size, sex ratio, age of patients, disease duration, and Glasgow Coma Scale [GSC]), details of interventions, and outcomes of interest. In addition, we extracted information related to methodological quality. Any disagreements were resolved by consulting a third senior investigator (Xiaoli Liu).

### Outcomes of interest

We defined the incidence of SAP as the primary outcome of this meta-analysis. In addition, we defined the incidence of aspiration and the length of hospital stay as the secondary outcomes. In this meta-analysis, we included dysphagia-caused aspiration, a common stroke sequela caused by bulbar palsy, as it increases the risk of SAP (29).

### Methodological quality

We assessed the methodological quality of included studies using the Cochrane Collaboration risk of bias tool (30), which was performed by two independent investigators (Qiao Fan and Xiaoli Liu). In this tool, the methodological quality assessment covers six domains, including selection bias (random sequence generation and allocation concealment), performance bias (blinding of personnel and participants), detection bias (blinding of outcome assessment), attrition bias (incomplete outcome data), reporting bias (selective reporting), and other bias. Briefly, the individual domain was labeled with high, unclear, or low risk, depending on whether a study was insufficient, unclear, or well-conducted with relevant information. Any disagreements were resolved by consulting a third senior investigator (Feng Guo) to achieve consensus.

### Statistical analysis

We expressed estimates of SAP and aspiration using risk ratio (RR) with the corresponding 95% CI. Meanwhile, the mean difference (MD) with the corresponding 95% CI was used to express the estimate of the length of hospital stay. We performed Chi-square test to determine whether there was heterogeneity across studies (31, 32). Then, the I^2^ statistic was used to quantify the level of statistical heterogeneity (33). Significant heterogeneity was considered to exist if a *p* value of <0.1 and an I^2^ of >50% were generated. We selected the random-effects model to calculate estimates regardless of the level of statistical heterogeneity, as differences between studies cannot be completely eliminated in a real setting (33). However, if significant statistical heterogeneity was detected, we performed a sensitivity analysis to check the robustness of the pooled results. For the length of hospital stay, we performed a subgroup analysis to calculate overall estimates based on stroke severity. Finally, we used Begg's and Egger's tests to check for publication bias if the cumulative number of eligible studies for individual outcomes exceeded ten (34). All statistical analyses were performed using Review Manager (RevMan) (version 5.4, the Nordic Cochrane Center, the Cochrane Collaboration, Copenhagen, 2014) (27), and publication bias examination was conducted using STATA 14.0 (State Corporation, Lake Way, Texas, USA). Statistical significance was described as *p* < 0.05.

### Trial sequential analysis

If estimates are repeated by introducing additional studies, the risk of false-positive results increases significantly. It's unreasonable to determine whether definitive conclusions can be drawn based on the conventional significance level (*p* < 0.05). Therefore, trial sequential analysis (TSA) was designed to help draw conclusions (35) by adjusting the conventional significance levels to construct trial sequential monitoring boundaries and estimate the required information size. A definitive conclusion can be drawn if the cumulative *Z*-curve crosses the trial sequential monitoring boundaries or the futility boundaries or the accumulated sample size was larger than the required information size. We defined a type I error of 5% and a statistical power of 90% to calculate the required information size. The incidence of SAP and aspiration was set at a relative risk reduction of 20% and the control incidence was calculated from the meta-analysis. For the length of hospital stay, mean difference and variance were calculated from empirical information. Heterogeneity was corrected using a model-based variance.

## Results

### Study selection

**Figure 1:**
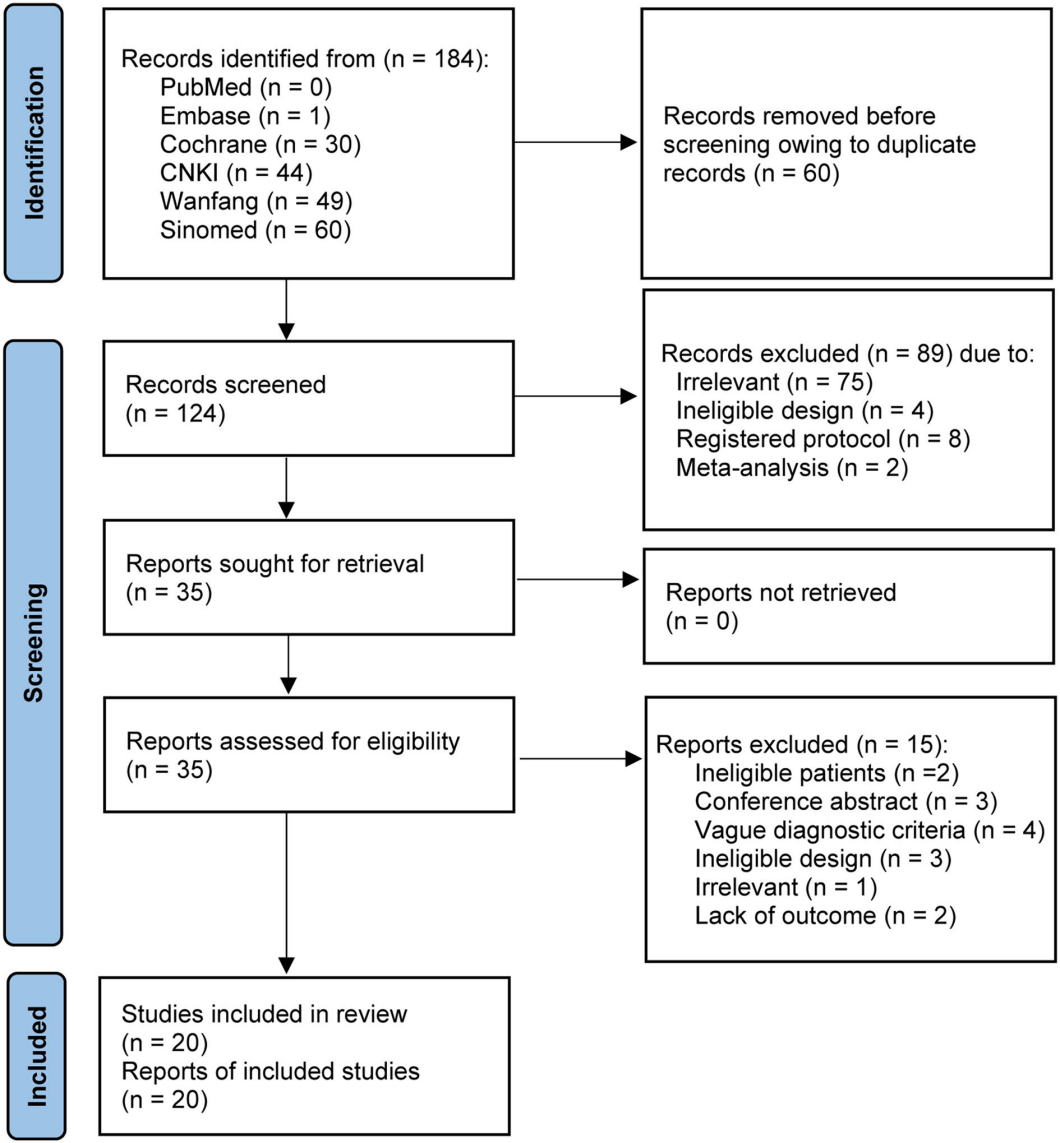
PRISMA flow chart of study identification and selection. CNKI, China National Knowledge Infrastructure. Sinomed, Chinese Biomedical Literature Database.

We retrieved 184 studies from five target databases using a sensitive search strategy. After the EndNote software automatically removed 60 duplicate studies, the titles and abstracts of the remaining 124 studies were screened, and 89 studies were further excluded. After a careful review of the full texts of the remaining 35 studies, 15 ineligible studies were excluded due to ineligible patients (*n* = 2), conference abstract (*n* = 3), vague diagnostic criteria (*n* = 4), ineligible design (*n* = 3), irrelevant topic (*n* = 1), and absence of outcome (*n* = 2). Ultimately, 20 studies (23–26, 36–51) were judged to meet the selection criteria. We created Figure 1 to depict the process of study retrieval and selection.

### Characteristics of studies

Table 1 summarizes the baseline characteristics of 20 eligible studies. All studies were published between 2011 and 2021. The sample size of each study ranged from 60 to 190, with a cumulative number of 1,916. Five studies (24, 39, 45, 48, 50) included only patients with severe strokes; however, only 2 studies (48, 50) reported GSC. All patients received standard stroke treatment, such as anticoagulant therapy and therapeutic strategy for improving cerebral circulation. None of the studies reported details of antibiotic use. The routine care protocols in all studies covered the essential elements (i.e., basic nursing, dietary nursing, and specialized nursing for neurological conditions), revealing comparability. All studies (23–26, 36–51) reported the incidence of SAP as the primary outcome. In addition, nine studies (37–41, 43, 46, 49, 50) reported the incidence of aspiration, and 9 studies (24, 25, 39, 41, 45, 47–50) reported the length of hospital stay as the secondary outcomes.

**Table 1:** Basic information of 20 eligible studies in the present meta-analysis.

**References**	**Country**	**Sample size, (male/female)**	**Age, years**	**Disease duration, d**	**GCS**	**Preventive interventions**
Zhao et al. (26)	China	62 (36/26) vs. 62 (34/28)	(68.9 ± 6.8) vs. (68.5 ± 7.1)	(11.7 ± 6.1) vs. (11 ± 5.1)	n.r.	Nasogastric tube maintenance, aseptic operation, body position change, assistance to expectorate sputum and clear foreign bodies, aerosol inhalation if necessary.
Xie (25)	China	43 (24/19) vs. 43 (25/18)	(66.4 ± 16.2) vs. (70.9 ± 12.8)	n.r.	n.r.	Nasogastric tube maintenance, oral care, assistance to expectorate sputum and clear foreign bodies.
Liu and Zhou (40)	China	41 (29/12) vs. 41 (27/14)	(43–88) vs. (45–89)	n.r.	n.r.	Feeding management, respiratory tract management, body position management.
Zhang (50)	China	60 (38/22) vs. 60 (35/25)	(64.2 ± 10.4) vs. (66.4 ± 11.3)	n.r.	(5.85 ± 1.45) vs. (6.12 ± 1.32)	Body position change, oral care, feeding management, assistance to expectorate sputum and clear foreign bodies.
Ding (37)	China	52 vs. 52 (64/40)	62.5 ± 2.4	n.r.	n.r.	Gastric tube maintenance, oral care, body position change, assistance to expectorate sputum and clear foreign bodies.
Ouyang (45)	China	30 (19/11) vs. 30 (18/12)	(62.3 ± 5.3) vs. (65.2 ± 5.2)	n.r.	n.r.	Gastric tube maintenance, oral care, body position change, assistance to expectorate sputum and clear foreign bodies.
Wu (48)	China	35 (19/16) vs. 35 (20/15)	(62 ± 8) vs. (63.5 ± 8.5)	n.r.	(5.71 ± 1.13) vs. (5.88 ± 1.36)	Position change, oral care, assistance to expectorate sputum and clear foreign bodies.
Sun et al. (47)	China	100 vs. 90	n.r.	n.r.	n.r.	Gastric tube maintenance, oral care, body position change, assistance to expectorate sputum and clear foreign bodies.
Huang (23)	China	50 (35/15) vs. 50 (36/14)	(61.5 ± 9.8) vs. (61.5 ± 9.8)	n.r.	n.r.	Gastric tube maintenance, oral care, body position change, respiratory tract management.
Lu and Zhu (42)	China	32 vs. 32 (41/23)	62-87	n.r.	n.r.	Gastric tube maintenance, oral care, body position change, assistance to expectorate sputum and clear foreign bodies.
Zhao et al. (51)	China	40 (32/8) vs. 30 (7/23)	(36–82) vs. (40–81)	n.r.	n.r.	Oral care, feeding management, body position management, and respiratory tract management.
Su (46)	China	63 (46/17) vs. (63 (44/19)	(40–81) vs. (41–80)	n.r.	n.r.	Gastric tube maintenance, oral care, body position management, assistance to expectorate sputum and clear foreign bodies.
Wu (49)	China	59 vs. 59 (64/54)	52.8 ± 4.2	n.r.	n.r.	Gastric tube maintenance, oral care, body position change, respiratory tract management.
Mou (24)	China	35 (32/3) vs. 35 (30/5)	(48.9 ± 5.6) vs. (46.5 ± 3.3)	n.r.	n.r.	Nasogastric tube maintenance, oral care, position change, assistance to expectorate sputum and clear foreign bodies.
Liu et al. (41)	China	72 (45/27) vs. 72 (41/31)	(65.0 ± 1.5) vs. (63.0 ± 2.5)	n.r.	n.r.	Nasogastric tube maintenance, oral care, body position change, assistance to expectorate sputum and clear foreign bodies.
Miao (44)	China	34 (21/13) vs. 34 (20/14)	(68.7 ± 6.1) vs. (67.8 ± 5.2)	n.r.	n.r.	Nasogastric tube maintenance, oral care, body position change, assistance to expectorate sputum and clear foreign bodies.
Meng (43)	China	40 (28/12) vs. 40 (27/13)	(66.6 ± 3.7) vs. (66.7 ± 3.7)	(5.8 ± 0.3) vs. (5.7 ± 0.3)	n.r.	Nasogastric tube maintenance, oral care, body position change, assistance to expectorate sputum and clear foreign bodies.
Liang (39)	China	55 (31/24) vs. 55 (33/22)	(65 ± 1.5) vs. (63 ± 2.5)	n.r.	n.r.	Nasogastric tube maintenance, aseptic operation, oral care, body position change, assistance to expectorate sputum and clear foreign bodies.
Jiang and Xu (38)	China	35 (21/14) vs. 35 (20/15)	(65.2 ± 7.7) vs. (65.0 ± 7.8)	n.r.	n.r.	Nasogastric tube maintenance, assistance to expectorate sputum and clear foreign bodies.
Chen et al. (36)	China	30 (16/14) vs. 30 (19/11)	(67.9 ± 8.4) vs. (66.8 ± 7.8)	n.r.	n.r.	Body position change, aseptic operation, assistance to expectorate sputum and clear foreign bodies.

GCS, Glasgow Coma Scale; SAP, stroke-associated pneumonia; AP, aspiration; LOH, length of hospital stay; n.r., not reported. For condition, both indicated inclusion of patients with ischemic and hemorrhagic strokes; n.a., not applicable.

### Risk of bias

**Figure 2:**
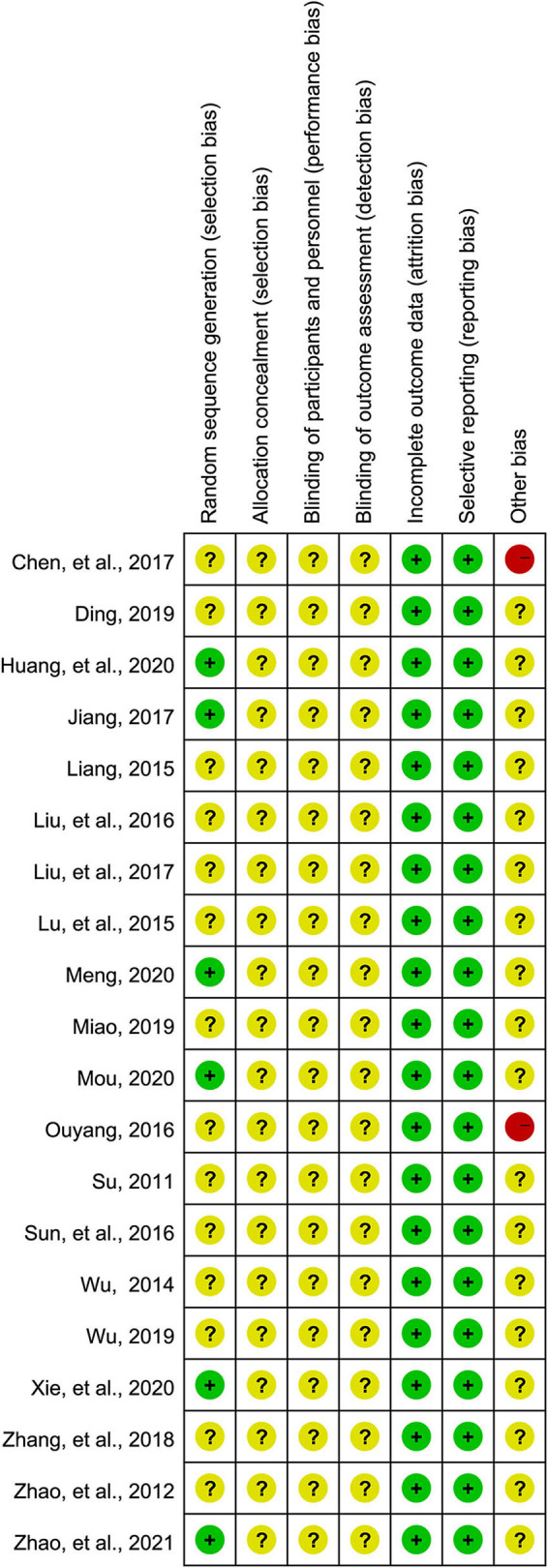
Risk of bias summary. Three colors including green, yellow, and red were used to represent the level of risk, which was corresponding to low, unclear, and high risk, respectively.

Although all eligible studies were described as RCTs, only 6 studies (23–26, 38, 43) described the methods for generating random sequences. What's more, it's unclear whether allocation concealment was conducted in all eligible studies due to inadequate information. Performance and detection bias were judged to be at unclear risk because none of the studies introduced methods for blinding personnel, participants, and outcome assessors. All studies were assessed as low risk for attrition and reporting biases. Two studies (36, 45) were rated as high risk for other biases due to the small sample size (≤30). The detailed bias risk assessment is summarized in Figure 2.

### Incidence of SAP

**Figure 3:**
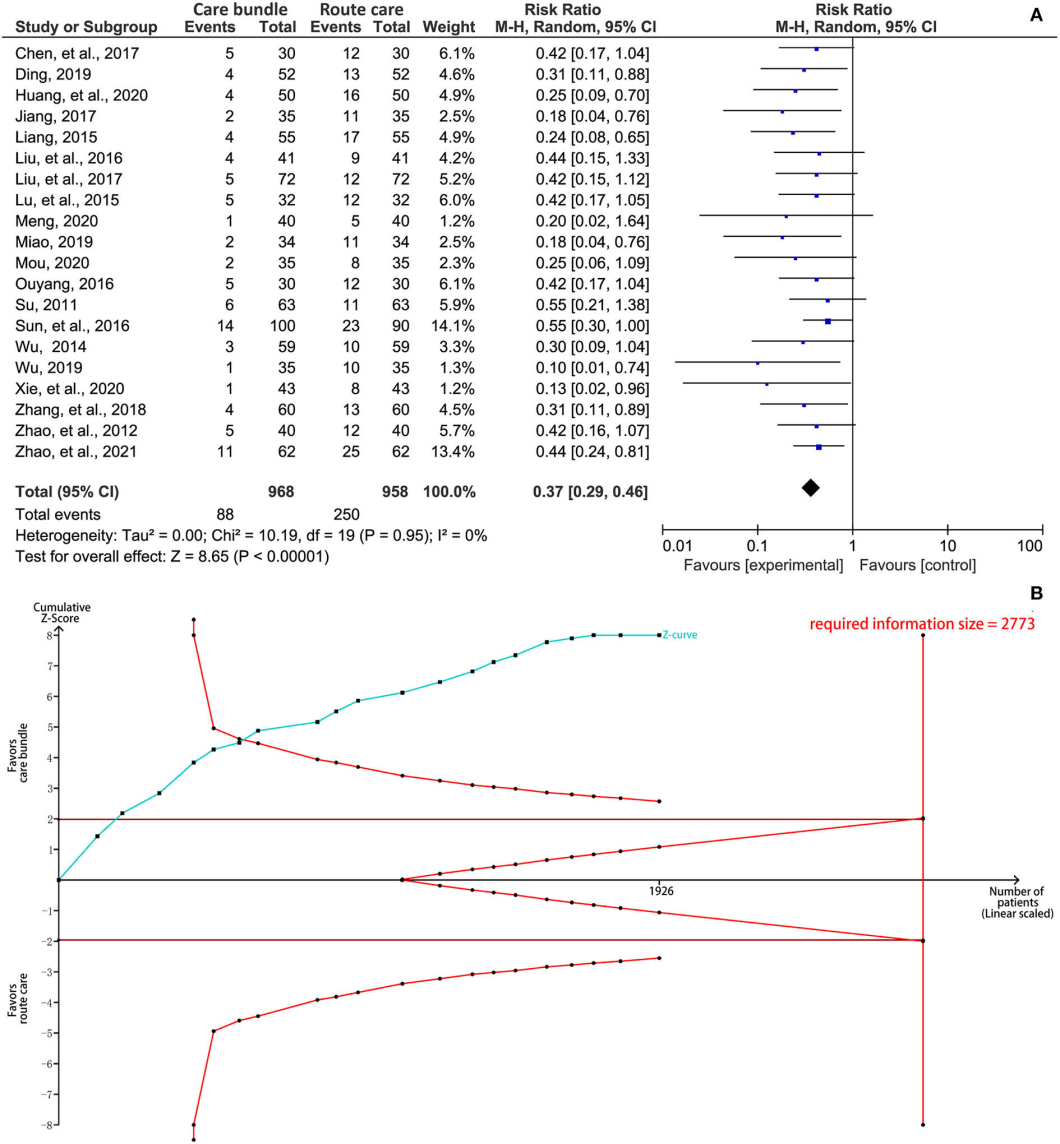
Synthesis analysis of the incidence of stroke-associated pneumonia. **(A)** forest plot and **(B)** results of TSA. CI, confidence interval; TSA, trial sequential analysis.

The meta-analysis showed that, compared with routine care protocol, the patient's care bundle was associated with a significantly lower incidence of SAP (RR, 0.37; 95%CI, 0.29–0.46; *p* < 0.01; Figure 3A), indicating a considerably preventive effect on the occurrence of SAP. Substantial statistical heterogeneity was not detected (I^2^ = 0%, *p* = 0.95). The result of the TSA revealed that the cumulative *Z*-curve crossed the trial sequential monitory boundary for benefit after the seventh study was added although the required information size of 2,773 was not achieved (Figure 3B), indicating that a definitive conclusion was drawn.

### Incidence of aspiration

**Figure 4:**
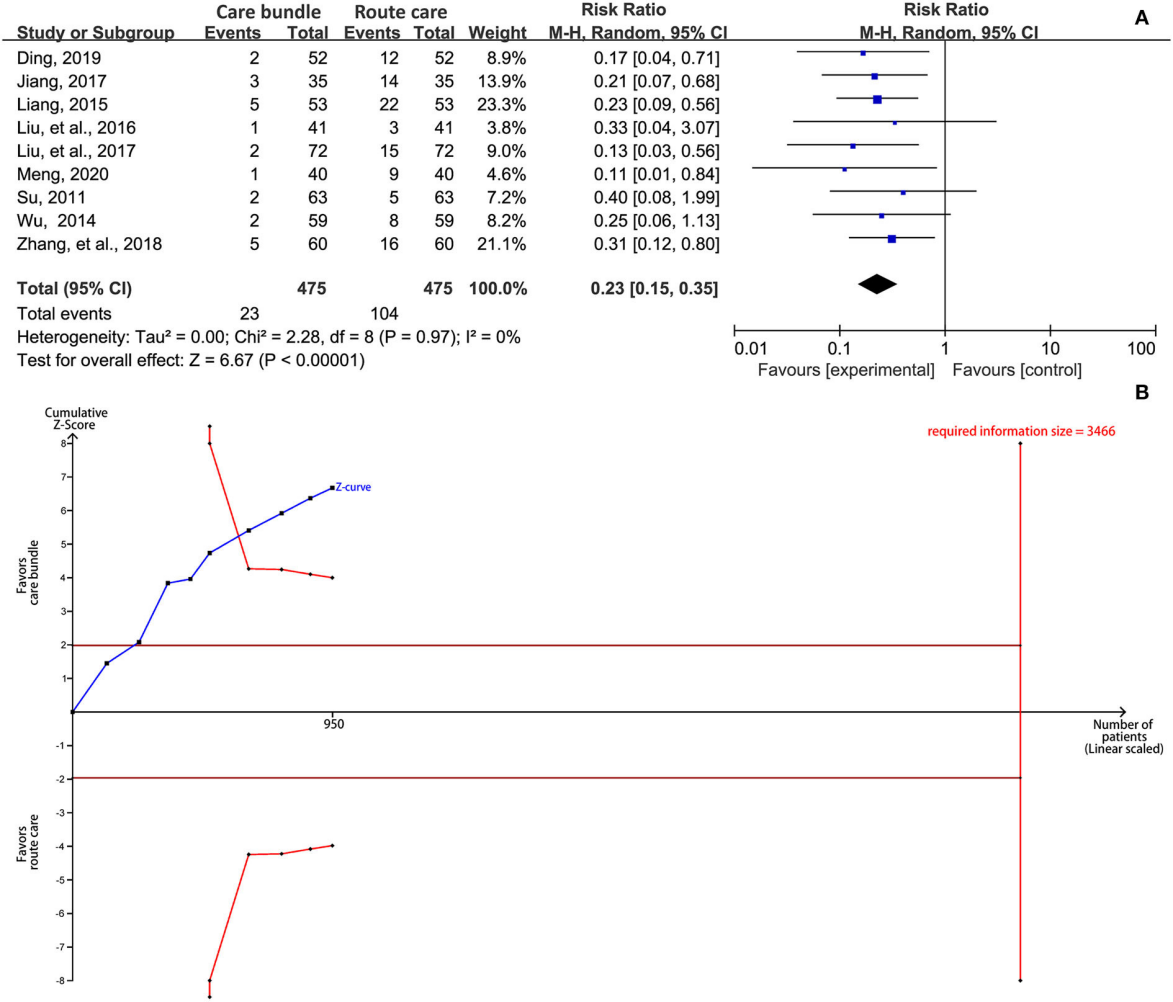
Synthesis analysis of the incidence of aspiration. **(A)** forest plot and **(B)** results of TSA. CI, confidence interval; TSA, trial sequential analysis.

The meta-analysis showed that patients who received a care bundle had a significantly lower aspiration compared with patients assigned to the routine care protocol (RR, 0.23; 95% CI, 0.15–0.35; *p* < 0.001; Figure 4A). No significant statistical heterogeneity was detected in this outcome (I^2^ = 0%, *p* = 0.97). The result from TSA suggested that cumulative Z-curve crossed the trial sequential monitoring boundary for benefit after the sixth study was added even though the required information size of 3,466 was not achieved (Figure 4B), indicating that no future study was required to validate this finding.

### Length of hospitalization

**Figure 5:**
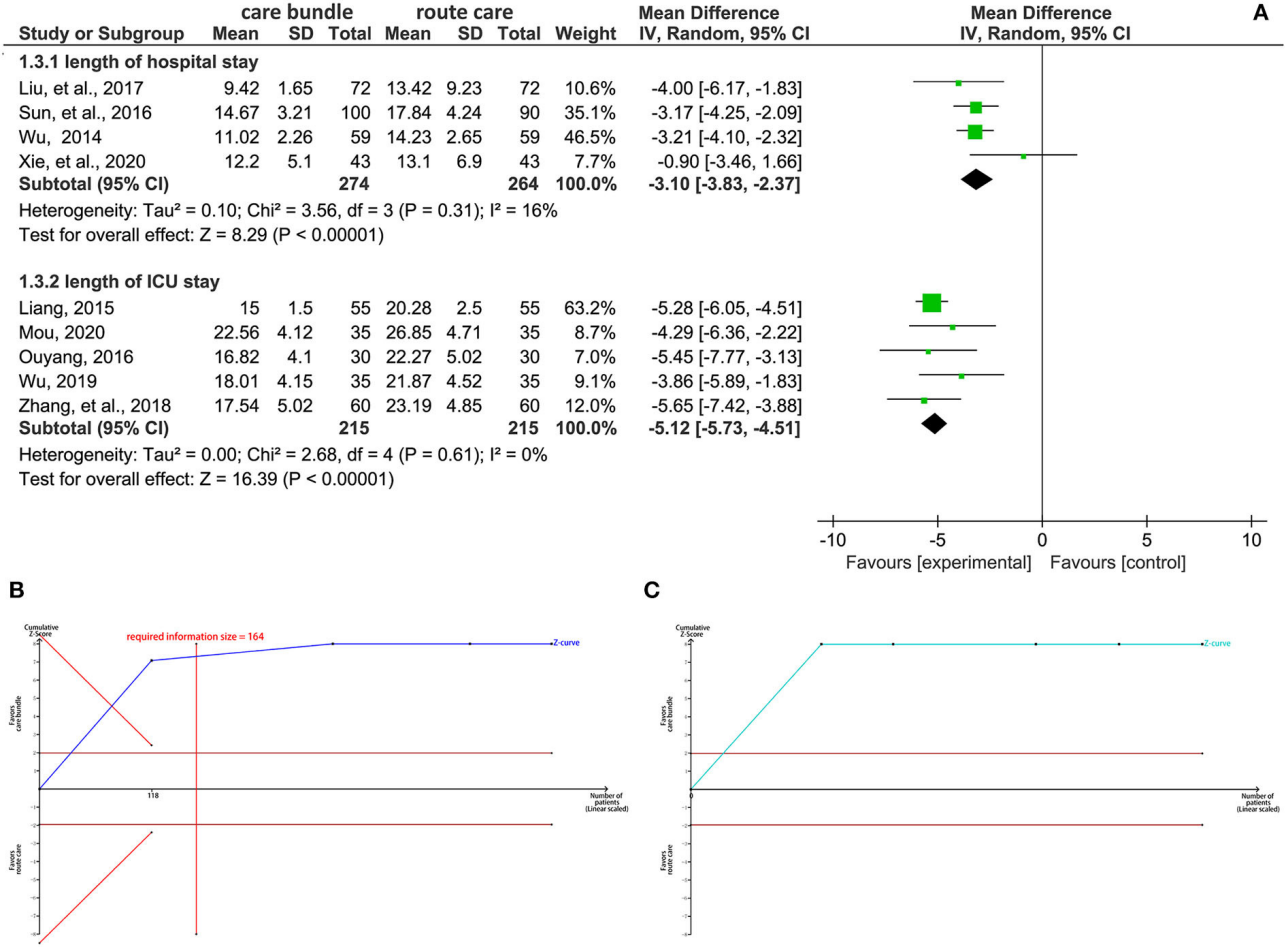
Synthesis analysis of the length of hospital stay. **(A)** forest plot **(B)** results of TSA for hospital stay **(C)** results of TSA for ICU stay. CI, confidence interval; TSA, trial sequential analysis.

We performed a subgroup analysis to determine whether the patient's care bundle could shorten the length of hospital stay based on stroke severity, which was determined using GSC scores. Four studies (25, 41, 47, 49) reported the length of hospital stay in patients with non-severe stroke, and the pooled result suggested that the care bundle significantly shortened the length of hospital stay compared with routine care protocol (MD, −3.10; 95% CI, −3.83 to −2.37; *p* < 0.001; I^2^ = 16%; Figure 5A). TSA indicated a definitive conclusion because the cumulative Z-curve crossed the trial sequential monitoring boundary for benefit and information threshold (required information size = 164) after the second study was added (Figure 5B). Five studies (24, 39, 45, 48, 50) reported the length of ICU stay for patients with severe stroke, and the pooled result suggested that the care bundle was associated with a significantly shortened length of ICU stay (MD, −5.12; 95% CI, −5.73 to −4.51; *p* < 0.001; I^2^ = 0%; Figure 5A). A definitive conclusion was drawn from TSA because the first information fraction exceeded 100% of the required information size when the first study was introduced (Figure 5C).

### Publication bias

**Figure 6:**
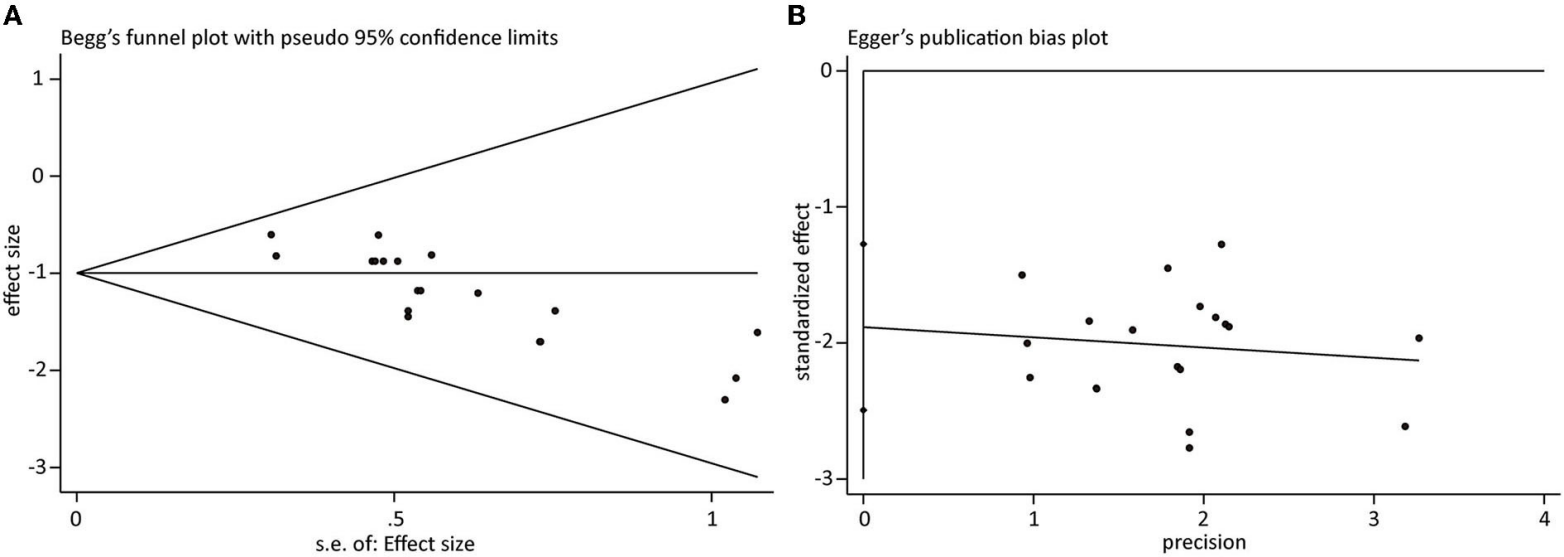
Publication bias examination based on **(A)** Begg's funnel plot and **(B)** Eggr's funnel plot.

For all outcomes, only the cumulative number of eligible studies reporting the incidence of SAP was greater than 10. Therefore, we performed the Begg's and Egger's tests to assess the risk of publication bias. As displayed in Figures 6A,B, an asymmetric plot was created for both Begg's test (z = 3.83, *p* < 0.001) and Egger's test (t = −6.48, *p* < 0.001), indicating the presence of publication bias.

## Discussion

To our knowledge, this is the first meta-analysis with trial sequential analysis to investigate the preventive effect of the patient's care bundle on SAP in stroke patients. Pooled results from 20 eligible RCTs involving 1,916 stroke patients suggested that, compared with routine care strategies, the patient's care bundle effectively prevented the occurrence of SAP and aspiration. Meanwhile, the patient's care bundle was also associated with the shortened length of hospitalization, especially the length of ICU stays among severe stroke patients.

SAP can be avoided through a variety of measures, such as organized stroke care. Sari et al. (52) reported a high incidence of SAP with severe neurological deficits on admission and dysphagia in a stroke care unit setting. In addition, Turner et al. (21) discovered that achieving a care bundle for ischemic stroke could significantly reduce the occurrence of SAP. It should be noted that the majority of currently available care strategies are single or multiple preventive interventions that are not evidence-based (22). Evidence-based protocol initiated by nurses for syndrome management is scientific, effective, targeted, safe, and systematic in stroke units (53). Patient's care bundle was first proposed by the Institute for Healthcare Quality Improvement (IHI) in 2001 and were subsequently defined as a nursing cluster involving 3–5 evidence-based, interrelated, simple, clear, and feasible practice measures (54). Specifically, the patient's care bundle provides further optimized nursing services for patients (54). Therefore, compared with an individual one of practice measures, a better effect will achieve for the treatment and recovery of patients after combining all measures (55). Actually, studies have suggested that accurate and effective implementation of the care bundle significantly reduced the risk of SAP and improved the functional prognosis in stroke patients (52, 56). Although some eligible studies indicated a comparable preventive effect of the care bundle with routine care strategies in the incidence of SAP and aspiration, it could not neglect that an insufficient sample size was included in these studies. Because of this reason, we conducted the TSA method to confirm that our findings could not be significantly reversed by future studies.

Several methodological advantages enhance the robustness and reliability of this meta-analysis. First, we systematically searched five databases for possible relevant studies, which greatly reduced the risk of missing eligible studies. Second, we selected the random-effects model to estimate results, which also greatly the reliability of pooled results. Third, Egger's and Begg's funnel plots were created to examine the risk of publication bias, greatly reducing the subjective error of visual inspection. Fourth, we introduced the TSA method to confirm the robustness of findings by creating adjusted monitoring boundaries, which significantly decreased the risk of drawing fallacious conclusions.

We must admit that some limitations compromise the reliability and robustness of our findings. First, we included 20 eligible RCTs for data analysis; however, each eligible study enrolled a small sample size. Therefore, the risk of fraudulent increases significantly. However, we conducted TSA to confirm the reliability of the conclusions. Second, the overall methodological quality was moderate due to insufficient explanation of methods of random sequence generation, and allocation concealment. Furthermore, there was no detailed description of allocation concealment or blinding of personnel, participants, and outcome assessors, which may have introduced some bias. Third, the applicability of our findings should be interpreted for other ethnic patients because all eligible studies were conducted in China. Fourth, although five databases were searched to identify 20 eligible studies, publication bias was still detected for the incidence of SAP. Nevertheless, we conducted TSA to decrease the risk of a false conclusion. Fifth, we did not register the formal protocol of this meta-analysis, which might bring some bias. However, we strictly developed an outline according to the recommendations made by the Cochrane handbook to possibly eliminate bias. Sixth, most eligible studies did not report stroke durations, which makes us could not investigate the impact of onset of stroke on interventional effects by introducing subgroup analysis. Moreover, these eligible studies did not report the type of units, staffing levels, mechanical thrombectomy, and thrombolysis, which might result in some biases. Finally, the effect of the antibiotics did not show in all eligible RCTs, so it may be a new direction of future research.

## Conclusion

Based on the results of 20 eligible RCTs, this meta-analysis suggests that the patient's care bundle is effective in preventing the occurrence of SAP and aspiration and shortening the length of hospital stay in stroke patients. However, it is necessary to design more high-quality studies to further validate our findings and investigate their applicability in other geographical regions.

## Data availability statement

The original contributions presented in the study are included in the article/Supplementary material, further inquiries can be directed to the corresponding author/s.

## Author contributions

FG: conceptualization and project administration. QF and XL: methodology. DS: software and supervision. FG and QF: validation, resources, writing original draft preparation, and visualization. FG and DS: formal analysis and writing review and editing. FG and XL: data curation. All authors have read and agreed to the published version of the manuscript.

## Conflict of interest

The authors declare that the research was conducted in the absence of any commercial or financial relationships that could be construed as a potential conflict of interest.

## Publisher's note

All claims expressed in this article are solely those of the authors and do not necessarily represent those of their affiliated organizations, or those of the publisher, the editors and the reviewers. Any product that may be evaluated in this article, or claim that may be made by its manufacturer, is not guaranteed or endorsed by the publisher.

## Abbreviations

SAP: stroke-associated pneumonia
AIS: acute ischemic stroke
RCTs: randomized controlled trials
PRISMA: Preferred Reporting Items for Systematic Reviews and Meta-Analyses
CNKI: China National Knowledge Infrastructure
Sinomed: Chinese Biological Medical Database
MeSH: medical subject heading
CT: computed tomography
MRI: magnetic resonance imaging
GSC: Glasgow Coma Scale
RR: risk ratio
CI: confidence interval
MD: mean difference
RevMan: Review Manager
TSA: trial sequential analysis
ICU: intensive care unit.
