# Supplementary material for: Patient's care bundle benefits to prevent stroke associated pneumonia: A meta-analysis with trial sequential analysis
Source: Front Neurol. 2022 Oct 31;13:950662. doi: 10.3389/fneur.2022.950662 (PMC9659564; doi:10.3389/fneur.2022.950662)
Supplement: Supplementary file 1 [file Table_1.DOCX]

**Search strategy of PubMed**

Search number Query Results

10 ((("Stroke"[Mesh]) OR ((((stroke[Title/Abstract]) OR (cerebrovascular accident[Title/Abstract])) OR (brain vascular accident[Title/Abstract])) OR (apoplexy[Title/Abstract]))) AND (("Pneumonia"[Mesh]) OR ((pneumonia[Title/Abstract]) OR (pneumonitis[Title/Abstract])))) AND (("Patient Care Bundles"[Mesh]) OR (((care bundle[Title/Abstract]) OR (cluster care[Title/Abstract])) OR (intensive nursing[Title/Abstract]))) - Schema: all 0

9 ("Patient Care Bundles"[Mesh]) OR (((care bundle[Title/Abstract]) OR (cluster care[Title/Abstract])) OR (intensive nursing[Title/Abstract])) 1,793

8 "Patient Care Bundles"[Mesh] 1,123

7 ((care bundle[Title/Abstract]) OR (cluster care[Title/Abstract])) OR (intensive nursing[Title/Abstract]) 932

6 ("Pneumonia"[Mesh]) OR ((pneumonia[Title/Abstract]) OR (pneumonitis[Title/Abstract])) 321,210

5 "Pneumonia"[Mesh] 236,544

4 (pneumonia[Title/Abstract]) OR (pneumonitis[Title/Abstract]) 152,272

3 ("Stroke"[Mesh]) OR ((((stroke[Title/Abstract]) OR (cerebrovascular accident[Title/Abstract])) OR (brain vascular accident[Title/Abstract])) OR (apoplexy[Title/Abstract])) 323,260

2 "Stroke"[Mesh] 155,063

1 (((stroke[Title/Abstract]) OR (cerebrovascular accident[Title/Abstract])) OR (brain vascular accident[Title/Abstract])) OR (apoplexy[Title/Abstract]) 285,070

**Search strategy of EMBASE**

.......................................................

No. Query Results Results

#10. #3 AND #6 AND #9 1

#9. #7 OR #8 2,505

#8. 'care bundle'/exp 1,660

#7. 'care bundle':ti,ab,kw OR 'cluster care':ti,ab,kw 1,722

OR 'intensive nursing':ti,ab,kw

#6. #4 OR #5 418,987

#5. 'pneumonia'/exp 367,718

#4. pneumonia:ti,ab,kw OR pneumonitis:ti,ab,kw 228,828

#3. #1 OR #2 558,624

#2. 'cerebrovascular accident'/exp 374,030

#1. stroke:ti,ab,kw OR 'cerebrovascular 457,917

accident':ti,ab,kw OR 'brain vascular

accident':ti,ab,kw OR apoplexy:ti,ab,kw

.......................................................

**Search strategy of Cochrane library**

ID Search Hits

#1 (stroke):ti,ab,kw OR (cerebrovascular accident):ti,ab,kw OR (brain vascular accident):ti,ab,kw OR (apoplexy):ti,ab,kw 63466

#2 MeSH descriptor: [Stroke] explode all trees 11029

#3 MeSH descriptor: [Stroke] explode all trees 11029

#4 #2 or #3 11029

#5 #1 or #4 63899

#6 (pneumonia):ti,ab,kw OR (pneumonitis):ti,ab,kw 18447

#7 MeSH descriptor: [Pneumonia] explode all trees 5114

#8 #6 or #7 19412

#9 (care bundle):ti,ab,kw OR (cluster care):ti,ab,kw OR (intensive nursing):ti,ab,kw 15332

#10 MeSH descriptor: [Patient Care Bundles] explode all trees 38

#11 #9 or #10 15335

#12 #5 and #6 and #11 in Trials 30
